# An Exostosis on the Root of the First Left Lower Molar

**Published:** 1853-07

**Authors:** Edwin Kreonele

**Affiliations:** Hyde Park, London.


### ARTICLE VIII.

*An Exostosis on the Root of the First Left Lower Molar.* By EDWIN KREONELE, Hyde Park, London.

THE following case of lengthened suffering produced by an exostosis on the root of the first left lower molar, came under my notice during the last summer. Since the extraction of the tooth, the gentleman has been quite free from pain in the mouth and inflammation of the eyes.

When ordering leeches, I did not suspect the presence of an exostosis, but thought the pain to arise from inflammation of the periosteum of the socket.

CASE.—J. M., a gentleman of twenty-five, consulted me for a pain in the face, arising from the first inferior molar of the left side. Upon examination, the tooth appeared slightly loose with considerable inflammation of the gum. I, therefore, objected to extract, but ordered leeches to the part, and a saline aperient at night. The following day he called again with the determination of having the tooth out. Having neglected the advice of the previous day, in consequence of a great dislike to leeches, he had not been free from pain for an hour.

Considerable resistance was offered to the removal of the tooth, but the cause was speedily made apparent, a small exostosis on the points of the roots. Immediate relief was obtained, which was permanent.

The history of this case is instructive. This tooth, together with several others, had been stopped some six years previously.

On removing the carious bone of this one, the membrane of the pulp cavity was exposed. The filling was consequently delayed until the membrane exposed could be caused to shrink away. For this purpose the strong spirit of camphor was used, and with complete success. Before the insertion of cement with which the tooth was filled, the opening into the pulp cavity was covered with a gold cap. The tooth now became quite useful, and for two or three years no uneasiness was ever experienced in it, after this period, however, when exposed to wet or other causes of cold, a grumbling pain was felt, and the gum more or less inflamed; when the symptoms were severe the gum was lanced, which generally afforded relief for the time. After about four years, after one of these attacks, the eyes became inflamed, especially the left, which continued so greatly to annoy him as to compel an entire cessation from business. His surgeon not knowing of the irritation of this tooth, treated the inflammation locally and constitutionally, but with very uncertain success, as he was still suffering from it when he called upon me; in addition to this, an abscess had formed, about four months before I saw him, in the lower third of the neck on the left side. The surgeon searched for a cause of irritation in vain, he suspected one of the teeth, but as his patient could see no connection between them and his eyes and neck, he did not tell him that occasionally he suffered in one that had been stopped, and as all that had been diseased were carefully stopped, he could not condemn any particular tooth. At last a change of air was ordered, and a trip to the continent was taken, and it was during this trip that, from exposure to wet, a severe cold was caught, which brought on a violent pain in this tooth, together with an aggravation of the inflammation of the eyes, which resulted in its extraction on his visit to London on his road home to the west.

Immediately after the extraction of the tooth the pain in the face ceased, the inflammation of the eyes became less, and abscess began to heal. In a short time he was, and has since continued, perfectly free from his disagreeable and painful companions.

A question naturally arises from this case; ought teeth to be filled after a part of the membrane has been exposed through decay, even when that part has, by the application of a tincture, been caused to shrink away so as to allow of the tooth's being stopped without giving the least pain in so doing?

In this case this treatment had been adopted; this result is before you, what should be the inference? or should one case in which such a train of evils followed, be allowed to greatly influence us? I have my own opinion on the point, and I think it one that merits the consideration of dentists.

